# Supplementary material for: The Efficiency of Pest Control Options against Two Major Sweet Corn Ear Pests in China
Source: Insects. 2023 Dec 6;14(12):929. doi: 10.3390/insects14120929 (PMC10743787; doi:10.3390/insects14120929)
Supplement: Supplementary file 1 [file insects-14-00929-s001.zip › Supplementary Figure S1.pdf]

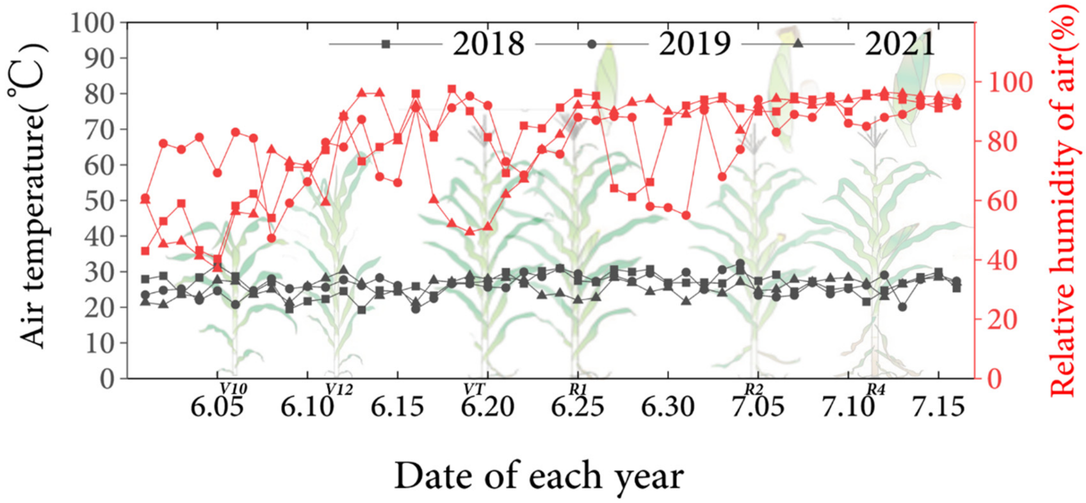

Figure S1. Temperature and humidity data of maize fields, the initial stage of ear pest occurrence near June 12th of each year. The average daily temperature is between 21-27°C; the average relative humidity is between 30 % -70 %, which has met the appropriate temperature and humidity for lepidopteran survival and reproduction, and such environmental conditions persisted throughout the silking phase and after.
